# Simulation-based learning in nephrology

**DOI:** 10.1093/ckj/sfae059

**Published:** 2024-03-12

**Authors:** Valentin Maisons, Antoine Lanot, Yosu Luque, Benedicte Sautenet, Emmanuel Esteve, Erwan Guillouet, Hélène François, Mickaël Bobot

**Affiliations:** Service de Néphrologie, CHU de Tours, Tours, France; U1246, INSERM, SPHERE, Université de Tours, Université de Nantes, Tours, Nantes, France, INI-CRCT, France; Normandie University, Unicaen, CHU de Caen Normandie, Nephrology, Côte de Nacre Caen, France; “ANTICIPE” U1086 INSERM-UCN, Centre Francois Baclesse, 3 Av. du General Harris, Caen, France; Soins Intensifs Néphrologiques Rein Aigu, Hôpital Tenon, APHP, Paris, France; Sorbonne Université, INSERM UMR_S1155, CORAKID, Hôpital Tenon, Paris, France; Service de Néphrologie, CHU de Tours, Tours, France; U1246, INSERM, SPHERE, Université de Tours, Université de Nantes, Tours, Nantes, France, INI-CRCT, France; Sorbonne Université, INSERM UMR_S1155, CORAKID, Hôpital Tenon, Paris, France; Service Néphrologie et Dialyses, Département de Néphrologie, Hôpital Tenon, APHP, Paris, France; Normandie University, Unicaen, CHU de Caen Normandie, Nephrology, Côte de Nacre Caen, France; NorSimS Simulation Center, Caen University Hospital, Caen, France; Sorbonne Université, INSERM UMR_S1155, CORAKID, Hôpital Tenon, Paris, France; Service de Transplantation rénale-Néphrologie, Département de néphrologie, Hôpital Pitié Salpétrière, APHP, Paris, France; Centre de Néphrologie et Transplantation Rénale, Hôpital de la Conception, AP-HM, Marseille, France; Aix Marseille Univ, INSERM 1263, INRAE 1260, C2VN, Marseille, France

**Keywords:** central venous catheter, kidney biopsy, nephrology, simulation, teaching

## Abstract

Simulation is a technique to replace and amplify real experiences with guided ones that evoke or replicate substantial aspects of the real world in a fully interactive fashion. In nephrology (a particularly complex specialty), simulation can be used by patients, nurses, residents, and attending physicians alike. It allows one to learn techniques outside the stressful environment of care such as central venous catheter placement, arteriovenous fistula management, learning about peritoneal dialysis, or performing a kidney biopsy. Serious games and virtual reality are emerging methods that show promise. Simulation could also be important in relational aspects of working in a team or with the patient. The development of simulation as a teaching tool in nephrology allows for maintaining high-quality training for residents, tailored to their future practice, and minimizing risks for patients. Additionally, this education helps nephrologists maintain mastery of technical procedures, making the specialty attractive to younger generations. Unfortunately, the inclusion of simulation training programmes faces occasional logistical or funding limitations that universities must overcome with the assistance and innovation of teaching nephrologists. The impact of simulation-based teaching on clinical outcomes needs to be investigated in clinical studies.

## Graphical Abstract

**Graphical Abstract:**
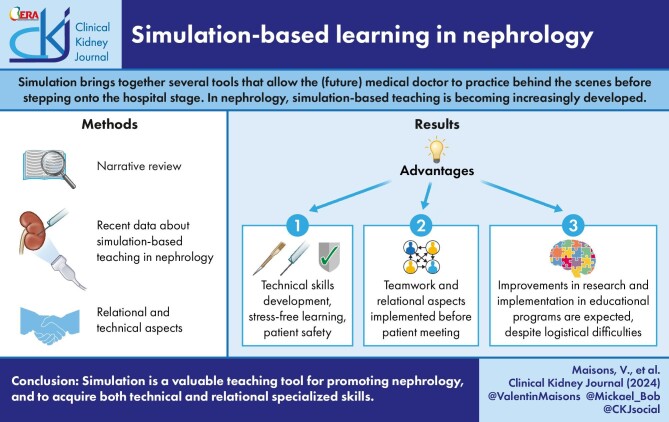

## INTRODUCTION

Nephrology is commonly perceived as a highly specialized and intricate medical discipline [1], demanding proficiency in technically challenging invasive procedures, including dialysis catheter placement and kidney biopsy (KB), alongside a comprehensive grasp of medical and scientific knowledge. Nephrology fellows are frequently exposed to chronically ill, polypathological patients, requiring advanced communication and clinical reasoning skills. Nephrology nurses also encounter specific tools and procedures, such as arteriovenous fistula (AVF) punctures, haemodialysis generators, apheresis, and peritoneal dialysis (PD) management. Currently, there is no standardized or universal approach for procedural training during nephrology fellowship, whereas it is commonly accepted that there is an important need for these specific techniques.

This necessitates the development of a new generation of pedagogical materials in nephrology, and simulation emerges as an apt methodology to address these challenges. Several calls for action and change have been published but, unfortunately, research is growing slowly in this field [2, 3].

Simulation is a technique to replace and amplify real experiences with guided ones, often ‘immersive’ in nature, that evoke or replicate substantial aspects of the real world in a fully interactive fashion [4]. The use of simulation-based teaching is well-suited to the training of healthcare students, as it enables learners to train in complete safety for the patient. It represents a real shift in the medical learning paradigm, moving from an era when technical gestures were taught in clinical practice, directly on the patient, to learning on a simulator. This approach to health education is ethically more acceptable.

Simulation enables the achievement of skills, procedures, knowledge, and cooperation between professionals. Simulation-based training in health professions education was shown to be consistently associated with significant effects on knowledge, skills, and behaviour outcomes in a meta-analysis [5].

Simulation training can be used in a wide variety of ways, depending on the simulation tool chosen. Procedural simulation is a set of learning technique in which simulators representing limb sections are used (AVF arms or ultrasound venipuncture steps) to acquire technical skills [6]. In immersive simulation, high-tech mannequins are used to reproduce clinical and paraclinical parameters, allowing scenarios to evolve and enabling teamwork. Immersive simulation offers a unique opportunity for multi-professional training, working on specific issues such as crisis resource management [7]. Multi-professional training improves professional communication, teamwork, and work efficiency, and opens up perspectives for the end of silo-based learning for healthcare players [8, 9]. Hybrid simulation can use procedural simulators and simulated patients to encourage learning of the technical gesture, while maintaining a relationship with the patient, and even working on empathy [10].

Simulation-based teaching could cover a wide range of fields in nephrology, from relational and technical aspects to teamwork, both in initial and continuing training of learners and professionals. Innovative learning tools, such as interactive numerical (and even social media-based) tools or ‘serious games’ involving high-fidelity mannequins or actors, are emerging and well-received by students [11–18]. These tools have the potential to enhance knowledge, clinical skills, and communication skills. Beyond nephrology fellows, several programmes address the specific training needs of nephrology nurses through gamification (escape game), high-fidelity mannequins for haemodialysis or PD, or AVF puncture simulators [19–22]. Dialysis Education Services, a board-certified dialysis technician training organization, and Lifeliqe, a global educational technology company, have jointly announced efforts to provide accessible programmes to address the shortage of dialysis staffing in the USA.

Beyond medical and paramedical training, several experiences have demonstrated the highly beneficial potential of simulation-based approaches to improve nephrology patients' independence [23].

### Central venous catheter placement

**Figure 1:**
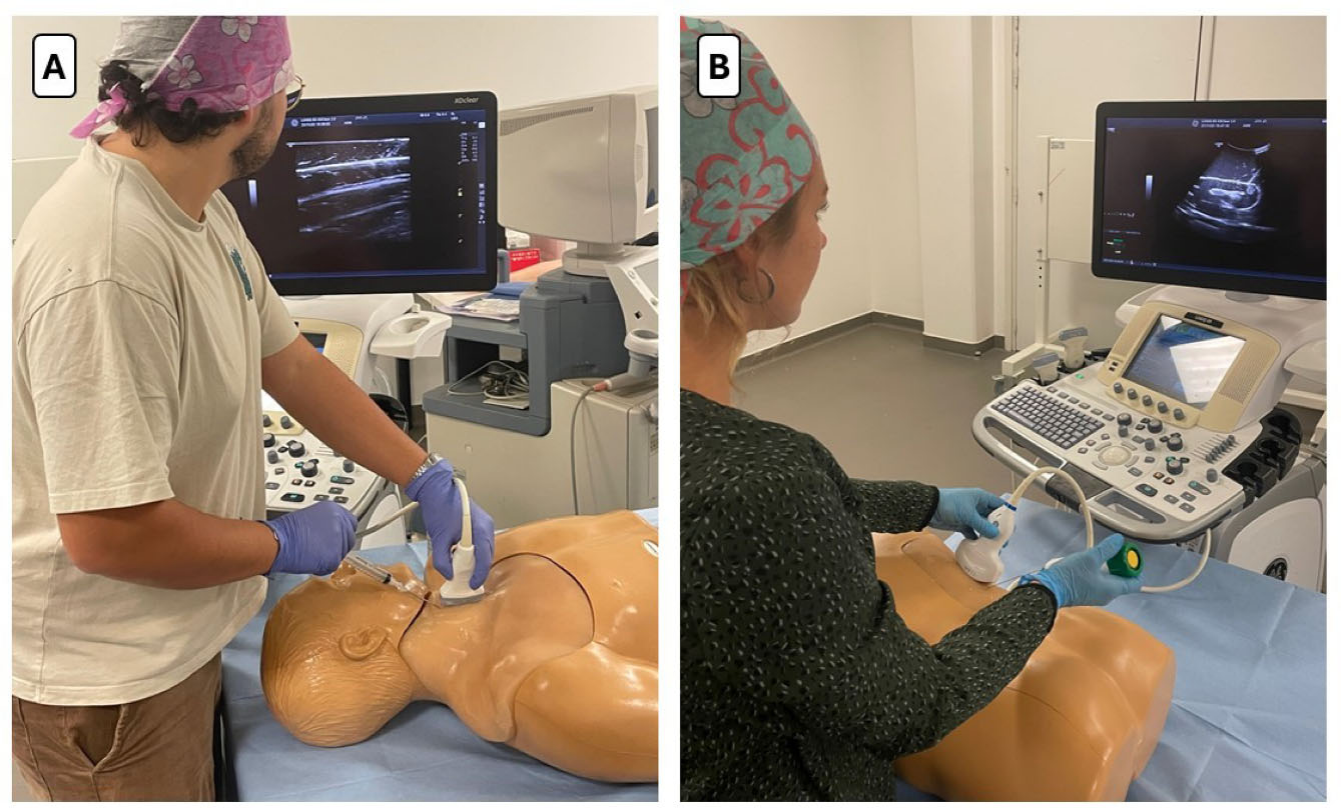
Simulation-based teaching of ultrasound-guided technical procedures on mannequins: (**A**) CVC placement and (**B**) KB.

The use of simulation techniques for training in central venous catheter (CVC) or haemodialysis catheter placement has developed in recent years, mainly in intensive care and anaesthesia. Their application is now extending into the field of nephrology. The simulation-based CVC insertion is probably the most studied to date. The first training methods have involved the use of turkey or pork meat and agar-agar pads with silicone tubes to simulate blood vessels [24]. These cost-effective solutions are gradually giving way to more realistic devices (Fig. 1a), notably through the use of 3D printing [25, 26]. It allows for the faithful reproduction of anatomical structures, including bones, arteries, and veins. In this case, the best reconstruction of the jugular and femoral regions is important for central haemodialysis catheters. Perfused human cadavers have also been used with the advantage of accurate anatomical fidelity [27].

So far, most guidelines recommend the insertion of CVC under ultrasound guidance [28]. Many studies confirmed the reduction in the number of attempts by venipuncture, failures, and main complications (arterial puncture, haematoma, haemothorax, or pneumothorax) related to the procedure. CVC insertion therefore requires appropriate imaging equipment and training. The hours spent in simulation training help to better understand how to use the ultrasound machine. During these privileged moments and outside stressful periods, the student can better understand standardized anatomy with the help of trained seniors.

Simulation approach could improve the technical proficiency of trainees and accelerate their skill development [29–32]. Recent advances in intensive care have shown that simulation can enhance fellows' proficiency in safely positioning and setting dialysis catheters, potentially reducing the risk of local complications [33–36]. In comparison to traditional training (supervised practice with real patients), mannequin-based training could lead to a reduction in catheter-related complications [30], such as catheter-related sepsis [31]. A meta-analysis of randomized trials seemed to confirm that simulation-based training for vascular access placement [37] (both arterial and venous) could improve overall success rates; however, it was unclear whether it could reduce adverse events, including mechanical complications. These results should be taken with caution because they suffer from problems of methodological standardization, in terms of learning and learner assessment. Improvement in learner confidence, satisfaction, and knowledge after simulation-based CVC training, evaluated by pre- versus post-intervention questionnaires and tests, are well documented, however [38].

Nephrology student-specific data concerning training for haemodialysis catheters placement, with a particular emphasis on the jugular approach, involved a limited number of participants (≤20 nephrology fellows) [33–35]. All these studies demonstrated significant enhancement in insertion scores through simulation-based learning when compared to conventional learning methods. Among them, a prospective observational cohort study assessed fellows’ skills performance between two groups: simulator trained fellows versus traditionally trained fellows [33]. The performance rates were significantly higher in the simulator trained fellows group (*P *< 0.001). Another study from the same team described a decrease in haemodialysis catheter insertion skills at 1 year while results remained stable at 6 months [35]. The reinforcement of skills for simulation training seems an important part to plan in teaching programmes. A retrospective cohort comparing fellows with or without simulation training for haemodialysis catheter placement reported data for 2481 catheter placed in 1787 patients. In the simulation versus traditional learning group respectively, less peri-procedure complications (8.3% vs. 11.2%, *P *= 0.02) and mechanical complications (1% vs. 2.4%, *P *= 0.02) during insertion were described [36].

A survey of French young nephrologists revealed that 82.2% of respondents recognized the value of teaching CVC placement through simulation [39]. These methods must be developed in collaboration with our colleagues trained in simulation methods in other specialties and with device manufacturers.

### Arteriovenous fistula

Several clinical practice guidelines have been published for the management of AVF, with recommendations about initiation of cannulation, preparation, technique, needle selection, surveillance, pain, and education [40]. Most of these subjects apply to nurses’ practices, or patients’ education for those treated with home haemodialysis. Simulation-based learning seems to be an ideal way of learning how to manage AVF. Such programmes could include learning clinical and ultrasound-based diagnosis to assess proper fistula function, strategy of cannulation, experimenting with the first AVF cannulation, and providing patients with information about their AVF. Echogenic, pulsatile, thrill-reproducing simulators exist and may be useful for procedural simulation sessions. However, data are scarce about simulation-based training concerning management of AVF. A Canadian team studied the impact of simulation-based learning in patients commencing training for nocturnal home haemodialysis on home visits, retraining, and technique survival. Twenty-eight patients who completed training using an innovation room (which simulates a patient's home) were compared with 21 historical matched controls. Patients who trained for home haemodialysis showed a trend towards needing less home visits, with no difference in the number of retraining session or technique survival [41]. An interventional multicentric study is currently underway in France to compare the number of adverse events related to AVF puncture performed by nurses who had received theoretical training plus simulation-based training and by a group of nurses who had received only theoretical training [19].

A model designed to represent the cubital fossa and proximal forearm was created to train vascular surgeons to the ultrasound-guided creation of a percutaneous AVF. Twenty vascular trainees and specialists were shown a video on creating a percutaneous AVF. A first group of 10 of them underwent supervised hands-on training on the model before performing a second rated percutaneous AVF creation on the model. The second group of 10 surgeons performed only a rated percutaneous AVF creation on the model. Based on the evaluation of a score rated by two observers, the study group participants increased their overall performance after training on the simulator [42].

Further studies will be necessary to evaluate the benefits of simulation-based training of nurses, nephrologists, and patients in AVF related care.

### Peritoneal dialysis

PD is an extra-renal purification treatment performed at home, either independently or with the assistance of nurses. Learning the technique is therefore necessary both for autonomous patients and for caregivers.

Few studies have addressed the issue of learning PD. data concerning the content of learning programmes and the most effective teaching methods are inconsistent [43–45]. International recommendations have been published by the International Society of Peritoneal Dialysis in 2016 [46]. It is recommended that teaching be carried out in an individualized way, in a manner appropriate to the learner's preferred style. The teaching methods described include a theoretical part based on different types of support, and a practical part, directly on the patient, using a mannequin or, more recently, virtually. The last two methods fall into the category of simulation-based learning. The so-called five-step method is advocated for teaching procedural skills [47]. According to this strategy, the steps of the dialysate exchange procedure are shown by the teacher and memorized, then performed on a mannequin by the learner, under supervision. Simulation is therefore part of a global teaching process according to the ISPD guidelines [46].

The use of mannequin to train (or re-train) patients or caregivers to perform the first dialysate exchanges is widespread, with several commercial systems available but no trial aimed to study the efficacy of using such devices. A virtual PD simulator was also developed by the Boston Children's Hospital to improve paediatric patients' independence [23]. Virtual reality tools have recently been developed in which the learner wears virtual reality goggles and gets in interaction with PD gear to virtually perform dialysate exchanges in real time [48]. A randomized controlled trial is planned to investigate the effectiveness of this virtual reality application.

Evidence for the best way to teach PD to patients and caregivers is lacking. Studies are needed to assess the effectiveness of training using simulation, considering outcomes such as patients’ and caregivers’ satisfaction, risk of PD related infection, or PD technique survival.

The assurance of having a functional peritoneal catheter is essential for successful PD. Most peritoneal catheters are placed by surgeons but there is a frequent shortfall in the peritoneal access surgery instruction during surgery residency. The Peritoneal Dialysis University for Surgeon is a course devised to provide didactic sessions and hands-on laboratory exercises. The catheter placement procedure is performed by surgeons using a high-fidelity human torso simulator. This training course using simulation-based learning was shown to produce long-term self-assessed improvement in surgical management and peritoneal catheter outcomes [49].

### Kidney biopsy

Percutaneous KB is a critically important tool for kidney disease diagnostic and is the most specific technical procedure for nephrologists. Training in performing KB is an important challenge in the teaching of nephrology. Indeed, in the USA, the learning of KB is declining and in a recent survey 65% of training graduates no longer perform KB and more than the half of nephrology programme directors consider that KB should not be required, mainly because of a lack of time for teaching and complicated logistics [50]. In France the observation is similar as only 34% of young nephrologists considered the training for KB sufficient, and 82% of them considered that performing KB is stressful [39]. Indeed, KB is feared by the nephrologists because of its associated bleeding risk, and the lack of opportunity to perform it regularly. Esposito *et al.* found that biopsies performed by trainees were associated with an equivalent rate of diagnostic efficiency and no more complications [51].

Various modalities have been used to train students in ultrasound-guided KB with high student adherence and clinical impact [39, 52–56]. Simulation models emerged in the 1990s, and nephrologists were inventive in simulating renal biopsy with low-cost homemade models: using, for example, pears in a box moved by an operator to mimic breathing [57], or boiled eggs in agar on ultrasound [58]. Simple models made of silicon or gelatine gels mimicking kidney anatomy on ultrasound were also developed [56, 59, 60]. The most described simulation model consists in performing an ultrasound-guided biopsy of a porcine or bovine kidney in a turkey or chicken breast [52, 55, 61] (approximate cost US$20 from the local butcher), or embedded into gelatine [54]. Those models allow to understand kidney structures on ultrasound, and allowed improvement in the confidence of trainees in KB [54, 55].

Rivera-Gorrin *et al.* also proposed an *in vivo* model in an anaesthetised pig, allowing the students to experience of more realistic experience with the resistance of the skin, the movement of kidney with breathing, and eventual complications (macroscopic haematuria, haematomas, AVF) [59].

More realistic models have since been developed. One of the most frequently used models today consists of a mannequin simulating a human trunk (Blue Phantom^TM^), into which an echogenic kidney phantom is inserted in an anatomical position (Fig. 1b). During a 4-hour workshop with both theorical teaching and training using this simulation model on a mannequin, all 21 participants showed a significant increased rate in knowledge and in perceived confidence. The impact on clinical performance was not assessed in this trial [56]. The advantages of these mannequins are their important reusability, and their realism for simulating an ultrasound-guided procedure close to a clinical situation involving a patient; however, their high price may be a limiting factor.

New generation models of KB using high-fidelity simulators and virtual reality are still under development [62, 63]. In a study on a specific virtual simulation tool for percutaneous KB, the training was rated highly with very positive subjective feedback but no data were provided on performance [64]. Advantages and disadvantages of the different simulation models of KB are summarized in Table 1.

**Table 1:** Advantages and disadvantages of simulators currently available for training in KB simulation.

Simulators	Advantages	Disadvantages	Cost	Realism
Homemade models (agar, gelatin…)	Cheap Portable	Limited realism Need preparation time Visible needle tracks after every use Sometimes disposable	+	+
Meat and animal organs	Realistic feel for tissue Cheap Portable	Short shelf life Storage and infection problems Need preparation time Ethical and ecological concerns	+	++
Mannequins	Portable Realistic No infection issues Large scanning surface Possible injection Long shelf life Reusable	Expensive Preformed Inability to integrate additional targets Visible needle tracks Non-tissue-like sensation	++	+++
Human of animal cadavers	Anatomical relevance Augmented realism	Availability Storage and infection concerns Ethical concerns	+++	+++
*In vivo* animal	Anatomical relevance Natural movements Management of complications	Availability Storage and infection concerns Expensive Ethical concerns	++++	++++
High-fidelity models and virtual reality	Augmented realism Possibility to generate specific scenarios Reusable	Expensive Lack of validation and experience	++++	+++

### Serious games and virtual reality

Playful aspects can also make a valuable contribution to simulation. Serious games are activities that combine a ‘serious’ intention—educational, informative, communicative, marketing, ideological, or training—with playful elements. Virtual reality refers to devices for the digital simulation of an environment by machine.

At present, these technologies are mainly used for patients in nephrology [65–67]. These interventions promote physical activity, cognitive stimulation, and social interaction. These two playful techniques are of interest in medical education, for rehearsing complex scenarios and improving social skills with patients or the care team. The physician of the 21st century must have obvious interpersonal skills. Augmented or virtual reality is also of procedural interest, being used in specialties such as neurosurgery, plastic surgery, urology, or dermatology [68–71]. It could also be used in interventional nephrology. Compared with experimentation on the patient or simulation on a mannequin, virtual reality offers a unique 3D anatomical vision, with the ability to decrypt the different levels.

The emergence of game-based learning modalities provides alternative approaches for teachers to improve the medical education process. In most cases, these educational formats are well-received by students and can create an immersive experience for learners, considered engaging, efficient, understandable, inspiring, and educational in comparison with traditional teaching activities [72].

### How to teach the patient–physician relationship with role play in nephrology

Simulation can also provide appropriate training methods to teach the patient–physician relationship, particularly in the setting of ‘breaking of bad news’. Indeed, very few studies have evaluated medical education and even fewer studies have properly assessed the teaching methods of the physician–patient relationship, especially during the breaking of bad news.

Traditionally, medical focus has been on diseases and organs rather than the physician–patient relationship. However, there has been a shift towards patient-centred care, emphasizing physician communication skills. This is crucial in fields such as oncology, where delivering bad news significantly affects patients [73]. In nephrology, severe chronic kidney disease is a life-altering diagnosis that can shatter patients’ life [74]. Improving communication through patient education and better training for nephrology trainees can reduce this impact [75]. Teaching communication skills and the delivery of bad news is often overlooked in medical education, requiring a re-evaluation of teaching practices [76]. Patient-centred care necessitates listening, empathy, and kindness, which are often considered innate and not taught. Our regional Health Authority funded a training programme on end-stage kidney disease diagnosis delivery, with strong support from CKD patients' associations.

Unlike several European countries, communication skills training has become a standard component of the curriculum in many medical schools in the USA. Additionally, numerous nephrology fellowships in the USA offer both formal and informal training in communication skills, including delivering difficult news. Much of the existing literature on this topic originates from the USA and, so far, only one of its kind was published in Europe [10]. Cohen *et al.* [18] conducted a study on communication skill courses for first-year US nephrology trainees, reporting positive outcomes. Participants in the day-long course demonstrated improved communication skills and more positive attitudes when discussing disease progression, dialysis therapy withdrawal, and end-of-life issues. In the USA, NephroTalk, a 3-day communication skills curriculum for nephrology fellows, significantly enhanced their ability to deliver bad news, leading to more effective patient encounters [77].

Our study [10], along with these findings, underscores the importance of incorporating communication skills training into the nephrology curriculum. We performed a single 4-hour session, using role play of several scenarios followed by debriefing sessions among the participants. Empathy in participants increased significantly after the training session and the improvement was sustained for several months afterwards. Our findings challenge the belief that senior practitioners are already adept at patient communication and suggests there is room for improvement in their communication skills and empathy. Also, they emphasize the teaching potential of simulation in nephrology [10]. This teaching is now compulsory for young nephrology residents in the Paris area.

Simulation serves various purposes in health professions education, has also been successfully employed to enhance interprofessional socialization [78, 79]. Simulation is particularly valuable when it comes to the skills required for delivering difficult news to patients and their families [80–82]. The diagnosis of end-stage kidney disease and the initiation of dialysis can be a traumatic experience for patients. As Hercz's analysis has shown [74], the transition to dialysis therapy is a challenging process that may lead to fragmented behaviour and thoughts. Therefore, we must begin shifting the culture of dialysis care delivery from a disease-centred approach, focused on clinical and laboratory factors, to a patient-centred approach that acknowledges the patients' need for support. It is well documented that the physician–patient relationship and the physician's empathy significantly affect patient care [73]. Physicians with greater empathy are more likely to establish better physician–patient relationships, resulting in higher patient satisfaction, treatment adherence, and improved health outcomes. Role-playing is an effective method for improving perspective-taking skills and the ability to empathize with patients, particularly in the nephrology field.

### Evaluation of simulation-based learning programmes in nephrology

As stated previously, pedagogical studies found that implementing simulation-based programmes for CVC placement can improve the learner's technical skills and decrease the complication rates. However, most of the studies on simulation-based learning in KB mainly evaluated student confidence and satisfaction but not post-teaching performance [56, 64].

Technical issues are not the only difficult part in nephrology and this speciality is often describe by students as a ‘complex and difficult courses’ [83]. Now simulation-based learning for clinical reasoning and treatment decision but also e-learning teaching tools take an important part in education improvement for nephrology students.

Interesting data on team learning exist in the nephrology field through an evaluation of a full day course using high-fidelity mannequins attended by 11 nephrology trainees and nine nurse specialists. The pre- and post-course assessments results showed improved knowledge scores of 56% to 72% (*P *< 0.05) and confidence scores to 57% to 71% (*P *< 0.005). The nephrology trainees reported at 1 year that the course improved their clinical practice and helped preparation for consultant roles [14]. Earlier in medical school, 103 undergraduate students taking part in a simulation training in nephrology agreed that simulation developed their clinical reasoning (87.4%) and decision-making skills (84.5%) [15].

In the acute kidney injury (AKI) field, a web-based, simulated AKI consult instrument has been created and validated with establishment of thresholds for remedial coaching. The pilot study included 15 fellows from four training programmes then 61 fellows from 20 training programmes [84]. The three following domains were assessed: problem representation, differential diagnosis, and diagnostic plan, and the intraclass correlation coefficients were 0.90, 0.70, and 0.50, respectively. The threshold for remedial coaching was met for 59% students in at least one domain.

Open teaching tools and social media are widely used by nephrology fellows overworld. NephMadness and NephSIM are the most famous but different learning possibilities are developed through social medias [12, 85–87]. Subjective assessments of these tools are very positive, but research data are missing.

### Development, future, and needs in nephrology

One key advantage of simulation is the opportunity for learners to engage in realistic, hands-on scenarios without engaging patient safety. Simulations provide a controlled environment where medical professionals can practice and refine their clinical skills, enhancing their competence and confidence. Additionally, simulation allows for the repetition of procedures and exposure to a variety of clinical situations, promoting skill mastery and improving decision-making under pressure. It also facilitates the integration of theoretical knowledge with practical application, bridging the gap between teach room and real-world patient care. Furthermore, simulation-based learning encourages teamwork and effective communication among healthcare providers. Interdisciplinary collaboration is fostered as teams work together to manage simulated patient scenarios, enhancing overall patient outcomes in clinical settings.

However, simulation-based learning also comes with limitations. High-fidelity simulation equipment is expensive to acquire and maintain, posing financial challenges for educational institutions. Besides, quality simulation-based training is performed in small groups and needs many teachers carrying supplementary logistic concerns to small universities or institutions. The consolidation of several universities or institutions may be necessary to carry out training, as well as a pooling of equipment that can be shared among various specialties. Indeed, certain procedural training sessions are common to multiple specialties, such as the placement of central venous catheters for nephrology and intensive care doctors.

Moreover, simulation is time-consuming for both teachers and learners. Clinical trials assessing the value of simulation-based learning on patient clinical outcomes are currently lacking. Such studies are difficult to carry out and require a long and precise follow-up but are definitely needed to validate the worthiness of simulation programmes to improve clinical outcomes.

For example, a key field for collaborating with intensive care teachers in simulation resources could be in the nephrological intensive care area. Indeed, point-of-care ultrasound education, managing cardiorespiratory arrest, or non-invasive ventilation, can be integrated into this teaching. The future probably involves shared simulation centres accommodating all specialties and rented for each specific training session. Industrial sponsors could collaborate with medical teaching institutions to mitigate financial burden and accompany the technical progresses they convey. One can also expect that the exponential progresses of conversational artificial intelligence and virtual reality environment deployment will help with enriching simulation possibilities and accessibility.

## CONCLUSION

**Figure 2:**
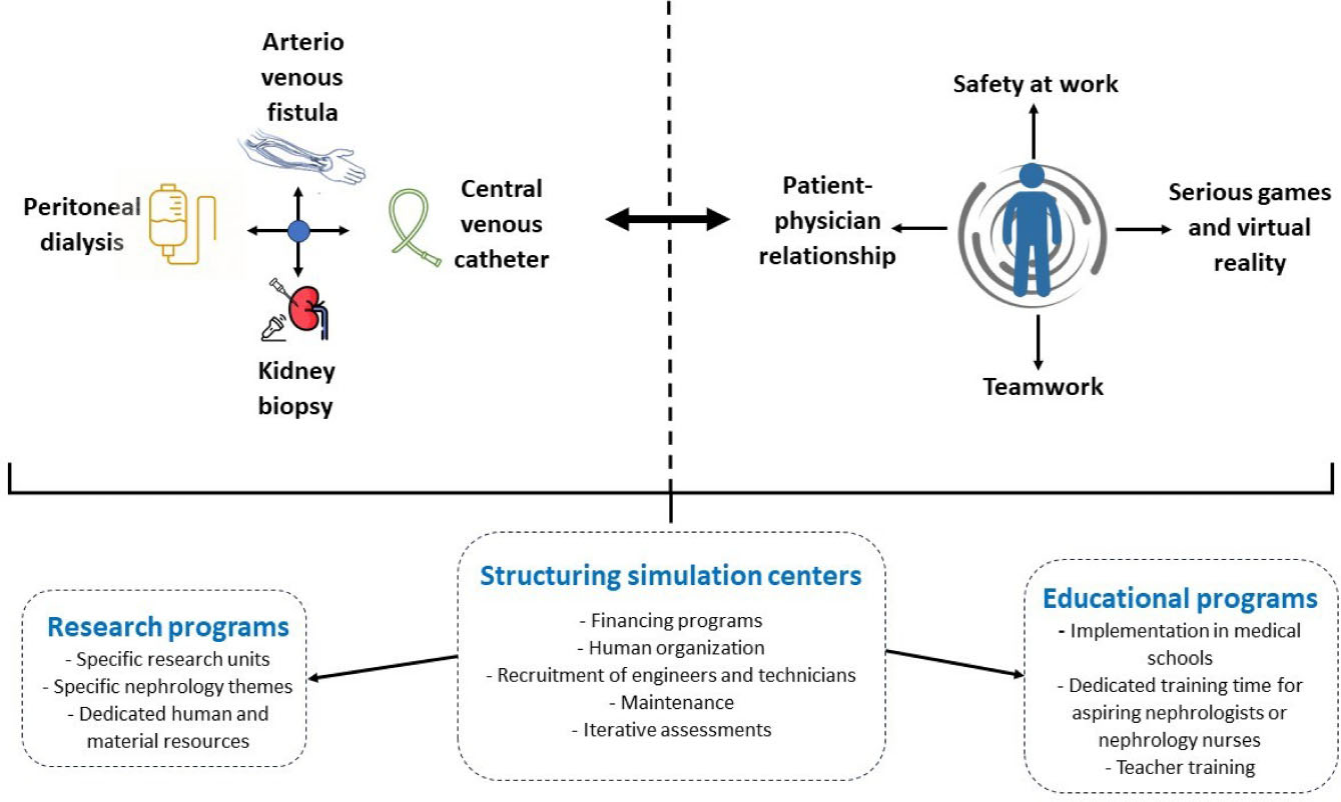
Implementing simulation-based learning in nephrology.

Despite some limitations, the advantages of simulation-based learning, including its ability to enhance safety, skill acquisition, and teamwork, underscore its valuable contribution to medical education and training. The development of simulation as a teaching tool in nephrology allows for maintaining high-quality training for residents, tailored to their future practice, and minimizing risks for patients. Additionally, this education helps nephrologists maintain mastery of technical procedures (e.g. KB), making the specialty attractive to younger generations. All arguments support the inclusion of these training programmes in the mandatory curriculum as shown in Fig. 2, but they face occasional logistical or funding limitations that universities must overcome with the assistance and innovation of teaching nephrologists and participation of industrial sponsors. The impact of simulation-based teaching on clinical outcomes needs to be investigated in clinical studies.

## Data Availability

No new data were generated or analysed in support of this research.
